# A second polymorph of aqua­(2,9-di­methyl-1,10-phenanthroline-κ^2^ *N*,*N*′)bis­(formato-κ*O*)copper(II)

**DOI:** 10.1107/S1600536808022812

**Published:** 2008-07-26

**Authors:** Jian-Li Lin, Wei Xu, Hong-Zhen Xie

**Affiliations:** a State Key Laboratory Base of Novel Functional Materials and Preparation Science, Faculty of Materials Science and Chemical Engineering, Ningbo University, Ningbo 315211, People’s Republic of China

## Abstract

A new monoclinic polymorphic form of the title compound, [Cu(HCO_2_)_2_(C_14_H_12_N_2_)(H_2_O)], is described. It differs from the first ortho­rhom­bic polymorph [Pan, Lin & Zheng (2005 ▶). *Z. Kristallogr. New Cryst. Struct.* **220**, 495–496] in the deviation of the Cu atom relative to the plane of the 2,9-dimethyl-1,10-phenanthroline (dmp) ligand. In the present structure, the Cu atom is shifted from the mean plane of the dmp ligand by only 0.005 (1) Å, compared with 0.318 (6) Å in the ortho­rhom­bic form. Hydrogen-bonding and π–π stacking inter­actions (mean inter­planar distance of 3.59 Å in the title compound) in the two different polymorphs are both essential to the supra­molecular assembly.

## Related literature

For the orthorhombic polymorph, see: Pan *et al.* (2005 ▶).
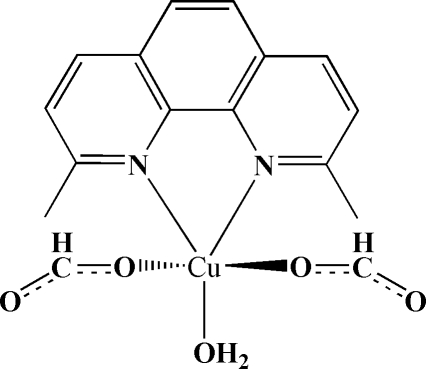


## Experimental

### 

#### Crystal data

[Cu(HCO_2_)_2_(C_14_H_12_N_2_)(H_2_O)]
*M* *_r_* = 379.85
Monoclinic, 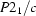

*a* = 10.669 (2) Å
*b* = 7.7677 (16) Å
*c* = 19.338 (4) Å
β = 94.22 (3)°
*V* = 1598.3 (6) Å^3^
*Z* = 4
Mo *K*α radiation
μ = 1.40 mm^−1^
*T* = 295 (2) K
0.26 × 0.17 × 0.09 mm

#### Data collection

Bruker P4 diffractometer
Absorption correction: multi-scan (*XSCANS*; Siemens, 1996 ▶) *T*_min_ = 0.749, *T*_max_ = 0.879
15099 measured reflections
3632 independent reflections
3202 reflections with *I* > 2σ(*I*)
*R*_int_ = 0.019
3 standard reflections every 97 reflections intensity decay: none

#### Refinement

*R*[*F* ^2^ > 2σ(*F* ^2^)] = 0.026
*wR*(*F* ^2^) = 0.072
*S* = 1.06
3632 reflections
219 parameters
H-atom parameters constrained
Δρ_max_ = 0.35 e Å^−3^
Δρ_min_ = −0.22 e Å^−3^

### 

Data collection: *XSCANS* (Siemens, 1996 ▶); cell refinement: *XSCANS*; data reduction: *XSCANS*; program(s) used to solve structure: *SHELXS97* (Sheldrick, 2008 ▶); program(s) used to refine structure: *SHELXL97* (Sheldrick, 2008 ▶); molecular graphics: *SHELXL97*; software used to prepare material for publication: *SHELXL97*.

## Supplementary Material

Crystal structure: contains datablocks global, I. DOI: 10.1107/S1600536808022812/fj2128sup1.cif

Structure factors: contains datablocks I. DOI: 10.1107/S1600536808022812/fj2128Isup2.hkl

Additional supplementary materials:  crystallographic information; 3D view; checkCIF report

## Figures and Tables

**Table:** 

Cu—O1	1.9450 (12)
Cu—O3	1.9546 (12)
Cu—O5	1.9726 (12)
Cu—N1	2.0328 (13)
Cu—N2	2.2801 (15)

**Table:** 

O1—Cu—O3	95.53 (6)
O1—Cu—O5	87.40 (6)
O1—Cu—N1	174.06 (6)
O1—Cu—N2	107.40 (6)
O3—Cu—O5	167.05 (6)
O3—Cu—N1	86.33 (5)
O3—Cu—N2	95.28 (6)
O5—Cu—N1	89.57 (5)
O5—Cu—N2	95.87 (5)
N1—Cu—N2	77.98 (6)

**Table 2:** Hydrogen-bond geometry (Å, °)

*D*—H⋯*A*	*D*—H	H⋯*A*	*D*⋯*A*	*D*—H⋯*A*
O5—H5*C*⋯O2^i^	0.88	1.86	2.714 (2)	166
O5—H5*B*⋯O4^ii^	0.89	1.72	2.605 (2)	175

Symmetry codes: (i) 
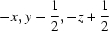
; (ii) 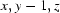.

**Fig. 1.**
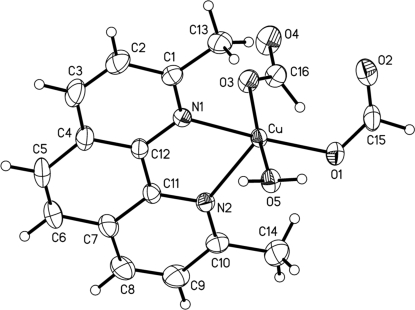
ORTEP view of the title compound. The displacement ellipsoids are drawn at 40% probability level.

**Fig. 2.**
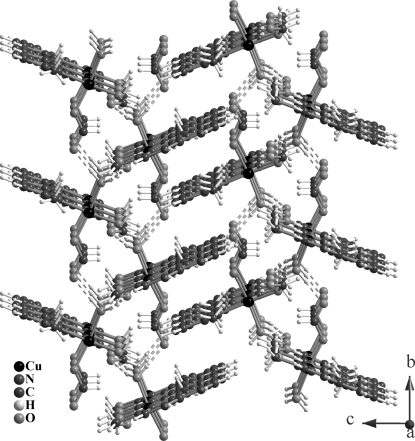
A perspective view of the crystal structure of (I), with hydrogen bonds shown as dashed lines.

## supplementary crystallographic information

### Comment

We reported a structure of the copper-dmp complex aqua-(2,9-dimethyl-1,10-phenanthroline-κ^2^*N*:*N*')-diromato-copper(II) previously, which crystallizes in space group Pna2_1_ (Pan, *et al.*,2005). On repeating the experiment recently, to our surprise, we found a new polyporph, (I), that had crystallized in the space group *P*2_1_/*c*.

The crystal structure of the title compound is very similar to the previously reported complex, built up by the [Cu(dmp)(H_2_O)(HCOO)_2_] complex molecules. The Cu atoms are each square pyramidally coordinated by two N atoms of one dmp ligand, three O atoms of two formate anions and one water molecule with the N2 atom of the dmp ligand at the apical position. The apical and basal Cu—N bond distances are 2.280 (1) and 2.033 (2) Å, respectively. The Cu—O bond distances to the formate anions are 1.945 (1) and 1.955 (1) Å, slightly longer than that to the water molecule (1.973 (1) Å). Suggesting that the formate anions possess better coordinating capability to the water molecule in the structure, which also show no significant difference from the isomer crystal structure that reported by us. The Cu atom is shifted by 0.153 (1) Å from the equatorial plane through N1, O1, O3 and O5 atoms towards the apical N2 atom. Through the intermolecular hydrogen bond the complex molecules are link into double chains with the chelating dmp ligands extending parallelly on one side along [010]. The substituted phenanthroline ligands of one double chain protrude into the grooves between adjacent aromatic planes of the neighboring double chain, yielding two-dimensional layers parallel to (100). It is found that the assembly of the double chains is due to interchain π-π stacking interactions between the dmp ligands (mean interplanar distance: 3.59 Å).

### Experimental

Dropwise addition of 2.0 ml (1.0 *M*) Na_2_CO_3_ to an aqueous solution of 0.075 g (0.442 mmol) CuCl_2_.2H_2_O in 5.0 ml H_2_O yielded pale blue deposit, which was separated by centrifugation and washed with doubly distilled water until no Cl^-^ anions are detectable in the supernatant. The precipitate was then added to a solution of 0.100 g (0.442 mmol) 2,9-dimethyl-1,10-phenanthroline in a mixed solvent consisting of 15 ml H_2_O and 15 ml me thanol. To the mixture 1.77 ml (1.0 *M*) formic acid was dropped and the precipitate was slowly dissolved under continuous stirring. The resulting blue solution was allowed to stand at room temperature, and slow evaporation for 10 days afforded blue plate crystals.

### Refinement

H atoms attached to C atoms of the dmp ligand were positioned geometrically and refined using a riding model, with C—H = 0.93 and 0.96 Å, and *U*_iso_(H) values set at 1.2 *U*eq(C) and 1.5 *U*eq(C), respectively. The H atoms of the water molecule and formate anions were located from difference Fourier maps.

### Crystal data

**Table:**

[Cu(HCO_2_)_2_(C_14_H_12_N_2_)(H_2_O)]	*F*_000_ = 780
*M**_r_* = 379.85	*D*_x_ = 1.579 Mg m^−^^3^
Monoclinic, *P*2_1_/*c*	Mo *K*α radiation λ = 0.71073 Å
Hall symbol: -P 2ybc	Cell parameters from 25 reflections
*a* = 10.669 (2) Å	θ = 5.0–12.5º
*b* = 7.7677 (16) Å	µ = 1.40 mm^−^^1^
*c* = 19.338 (4) Å	*T* = 295 (2) K
β = 94.22 (3)º	Plate, blue
*V* = 1598.3 (6) Å^3^	0.26 × 0.17 × 0.09 mm
*Z* = 4	

### Data collection

**Table:**

Bruker P4 diffractometer	*R*_int_ = 0.019
Radiation source: fine-focus sealed tube	θ_max_ = 27.5º
Monochromator: graphite	θ_min_ = 3.3º
*T* = 295(2) K	*h* = −13→13
θ/2θ scans	*k* = −10→9
Absorption correction: multi-scan(XSCANS; Siemens, 1996)	*l* = −24→25
*T*_min_ = 0.749, *T*_max_ = 0.879	3 standard reflections
15099 measured reflections	every 97 reflections
3632 independent reflections	intensity decay: none
3202 reflections with *I* > 2σ(*I*)	

### Refinement

**Table:**

Refinement on *F*^2^	Secondary atom site location: difference Fourier map
Least-squares matrix: full	Hydrogen site location: inferred from neighbouring sites
*R*[*F*^2^ > 2σ(*F*^2^)] = 0.026	H-atom parameters constrained
*wR*(*F*^2^) = 0.072	*w* = 1/[σ^2^(*F*_o_^2^) + (0.0413*P*)^2^ + 0.5201*P*] where *P* = (*F*_o_^2^ + 2*F*_c_^2^)/3
*S* = 1.06	(Δ/σ)_max_ = 0.001
3632 reflections	Δρ_max_ = 0.35 e Å^−^^3^
219 parameters	Δρ_min_ = −0.22 e Å^−^^3^
Primary atom site location: structure-invariant direct methods	Extinction correction: none

### Special details

**Table:**

Geometry. All e.s.d.'s (except the e.s.d. in the dihedral angle between two l.s. planes) are estimated using the full covariance matrix. The cell e.s.d.'s are taken into account individually in the estimation of e.s.d.'s in distances, angles and torsion angles; correlations between e.s.d.'s in cell parameters are only used when they are defined by crystal symmetry. An approximate (isotropic) treatment of cell e.s.d.'s is used for estimating e.s.d.'s involving l.s. planes.
Refinement. Refinement of *F*^2^ against ALL reflections. The weighted *R*-factor *wR* and goodness of fit *S* are based on *F*^2^, conventional *R*-factors *R* are based on *F*, with *F* set to zero for negative *F*^2^. The threshold expression of *F*^2^ > σ(*F*^2^) is used only for calculating *R*-factors(gt) *etc*. and is not relevant to the choice of reflections for refinement. *R*-factors based on *F*^2^ are statistically about twice as large as those based on *F*, and *R*- factors based on ALL data will be even larger.

### Fractional atomic coordinates and isotropic or equivalent isotropic displacement parameters (Å^2^)

**Table:**

	*x*	*y*	*z*	*U*_iso_*/*U*_eq_	
Cu	0.209657 (18)	0.90493 (2)	0.156116 (9)	0.02440 (7)	
N1	0.21922 (12)	0.80402 (17)	0.05966 (6)	0.0257 (3)	
N2	0.42113 (13)	0.89494 (17)	0.14606 (7)	0.0275 (3)	
C1	0.11886 (17)	0.7632 (2)	0.01841 (9)	0.0325 (4)	
C2	0.1317 (2)	0.6999 (3)	−0.04910 (10)	0.0472 (5)	
H2A	0.0605	0.6741	−0.0778	0.057*	
C3	0.2471 (2)	0.6765 (3)	−0.07224 (10)	0.0508 (5)	
H3A	0.2552	0.6332	−0.1165	0.061*	
C4	0.35505 (19)	0.7178 (3)	−0.02912 (9)	0.0393 (4)	
C5	0.4803 (2)	0.6986 (3)	−0.04949 (11)	0.0541 (6)	
H5A	0.4929	0.6540	−0.0931	0.065*	
C6	0.5804 (2)	0.7435 (3)	−0.00711 (11)	0.0537 (6)	
H6A	0.6609	0.7298	−0.0217	0.064*	
C7	0.56433 (17)	0.8120 (3)	0.05998 (10)	0.0397 (4)	
C8	0.66470 (19)	0.8661 (3)	0.10598 (12)	0.0511 (5)	
H8A	0.7468	0.8577	0.0931	0.061*	
C9	0.64241 (19)	0.9302 (3)	0.16884 (12)	0.0478 (5)	
H9A	0.7091	0.9659	0.1992	0.057*	
C10	0.51845 (17)	0.9430 (2)	0.18838 (9)	0.0350 (4)	
C11	0.44274 (15)	0.8321 (2)	0.08260 (8)	0.0289 (3)	
C12	0.33615 (16)	0.7831 (2)	0.03694 (8)	0.0281 (3)	
C13	−0.00801 (18)	0.7856 (3)	0.04536 (10)	0.0438 (4)	
H13A	−0.0302	0.6831	0.0694	0.066*	
H13B	−0.0691	0.8066	0.0073	0.066*	
H13C	−0.0062	0.8816	0.0767	0.066*	
C14	0.4920 (2)	1.0068 (3)	0.25888 (10)	0.0537 (6)	
H14A	0.4243	1.0884	0.2547	0.081*	
H14B	0.5659	1.0614	0.2802	0.081*	
H14C	0.4691	0.9116	0.2870	0.081*	
O1	0.18364 (13)	0.98991 (18)	0.24857 (6)	0.0408 (3)	
O2	−0.02000 (15)	1.0452 (3)	0.22488 (8)	0.0613 (4)	
C15	0.0760 (2)	1.0407 (3)	0.26275 (10)	0.0471 (5)	
O3	0.18495 (13)	1.12747 (15)	0.10994 (6)	0.0365 (3)	
O4	0.18327 (18)	1.41073 (16)	0.10823 (8)	0.0530 (4)	
C16	0.19793 (18)	1.2723 (2)	0.13792 (9)	0.0360 (4)	
O5	0.19568 (12)	0.67025 (15)	0.19376 (6)	0.0344 (3)	
H5B	0.1958	0.5834	0.1636	0.051*	
H5C	0.1416	0.6452	0.2242	0.054*	
H15	0.0751	1.0904	0.3111	0.052*	
H16	0.2383	1.2705	0.1852	0.049*	

### Atomic displacement parameters (Å^2^)

**Table:**

	*U*^11^	*U*^22^	*U*^33^	*U*^12^	*U*^13^	*U*^23^
Cu	0.02683 (11)	0.02277 (11)	0.02410 (11)	0.00161 (7)	0.00524 (7)	−0.00126 (7)
N1	0.0280 (7)	0.0227 (6)	0.0263 (6)	−0.0003 (5)	0.0021 (5)	−0.0001 (5)
N2	0.0267 (7)	0.0283 (7)	0.0276 (6)	−0.0012 (5)	0.0032 (5)	0.0011 (5)
C1	0.0353 (9)	0.0304 (8)	0.0313 (8)	−0.0023 (7)	−0.0011 (7)	−0.0003 (7)
C2	0.0495 (12)	0.0554 (12)	0.0350 (9)	−0.0055 (10)	−0.0086 (8)	−0.0096 (9)
C3	0.0627 (14)	0.0601 (13)	0.0297 (9)	−0.0010 (11)	0.0040 (9)	−0.0158 (9)
C4	0.0467 (11)	0.0421 (10)	0.0300 (8)	0.0022 (8)	0.0093 (7)	−0.0060 (8)
C5	0.0587 (14)	0.0690 (15)	0.0373 (10)	0.0073 (11)	0.0227 (9)	−0.0110 (10)
C6	0.0430 (12)	0.0737 (15)	0.0474 (11)	0.0080 (10)	0.0234 (9)	−0.0016 (11)
C7	0.0319 (9)	0.0479 (11)	0.0408 (9)	0.0023 (8)	0.0126 (7)	0.0052 (9)
C8	0.0246 (9)	0.0722 (14)	0.0575 (12)	−0.0005 (9)	0.0102 (8)	0.0079 (11)
C9	0.0306 (10)	0.0592 (13)	0.0526 (12)	−0.0096 (9)	−0.0033 (8)	0.0047 (10)
C10	0.0323 (9)	0.0346 (9)	0.0374 (9)	−0.0041 (7)	−0.0018 (7)	0.0021 (7)
C11	0.0292 (8)	0.0288 (8)	0.0295 (7)	0.0007 (6)	0.0077 (6)	0.0020 (7)
C12	0.0327 (8)	0.0264 (7)	0.0258 (7)	0.0020 (6)	0.0070 (6)	0.0006 (6)
C13	0.0308 (9)	0.0564 (12)	0.0434 (10)	−0.0056 (9)	−0.0033 (8)	0.0005 (9)
C14	0.0484 (12)	0.0687 (15)	0.0426 (11)	−0.0057 (11)	−0.0066 (9)	−0.0160 (11)
O1	0.0475 (8)	0.0463 (8)	0.0293 (6)	0.0104 (6)	0.0073 (5)	−0.0055 (6)
O2	0.0465 (9)	0.0904 (13)	0.0485 (8)	0.0135 (9)	0.0128 (7)	−0.0072 (9)
C15	0.0577 (13)	0.0534 (12)	0.0319 (9)	0.0127 (10)	0.0159 (9)	−0.0061 (9)
O3	0.0529 (8)	0.0249 (6)	0.0319 (6)	0.0037 (5)	0.0045 (5)	0.0000 (5)
O4	0.0859 (12)	0.0259 (7)	0.0479 (8)	0.0012 (7)	0.0098 (8)	0.0004 (6)
C16	0.0443 (10)	0.0292 (9)	0.0346 (8)	−0.0012 (7)	0.0028 (7)	−0.0013 (7)
O5	0.0415 (7)	0.0271 (6)	0.0359 (6)	−0.0028 (5)	0.0122 (5)	0.0032 (5)

### Geometric parameters (Å, °)

**Table:**

Cu—O1	1.9450 (12)	C7—C11	1.408 (2)
Cu—O3	1.9546 (12)	C8—C9	1.351 (3)
Cu—O5	1.9726 (12)	C8—H8A	0.9300
Cu—N1	2.0328 (13)	C9—C10	1.405 (3)
Cu—N2	2.2801 (15)	C9—H9A	0.9300
N1—C1	1.326 (2)	C10—C14	1.497 (3)
N1—C12	1.363 (2)	C11—C12	1.439 (2)
N2—C10	1.327 (2)	C13—H13A	0.9600
N2—C11	1.356 (2)	C13—H13B	0.9600
C1—C2	1.411 (2)	C13—H13C	0.9600
C1—C13	1.496 (3)	C14—H14A	0.9600
C2—C3	1.353 (3)	C14—H14B	0.9600
C2—H2A	0.9300	C14—H14C	0.9600
C3—C4	1.408 (3)	O1—C15	1.264 (2)
C3—H3A	0.9300	O2—C15	1.215 (3)
C4—C12	1.403 (2)	C15—H15	1.0122
C4—C5	1.429 (3)	O3—C16	1.252 (2)
C5—C6	1.343 (3)	O4—C16	1.223 (2)
C5—H5A	0.9300	C16—H16	0.9821
C6—C7	1.424 (3)	O5—H5B	0.8914
C6—H6A	0.9300	O5—H5C	0.8760
C7—C8	1.405 (3)		
			
O1—Cu—O3	95.53 (6)	C9—C8—H8A	119.9
O1—Cu—O5	87.40 (6)	C7—C8—H8A	119.9
O1—Cu—N1	174.06 (6)	C8—C9—C10	119.97 (19)
O1—Cu—N2	107.40 (6)	C8—C9—H9A	120.0
O3—Cu—O5	167.05 (6)	C10—C9—H9A	120.0
O3—Cu—N1	86.33 (5)	N2—C10—C9	121.54 (18)
O3—Cu—N2	95.28 (6)	N2—C10—C14	117.62 (17)
O5—Cu—N1	89.57 (5)	C9—C10—C14	120.81 (18)
O5—Cu—N2	95.87 (5)	N2—C11—C7	122.89 (16)
N1—Cu—N2	77.98 (6)	N2—C11—C12	118.11 (14)
C1—N1—C12	119.71 (14)	C7—C11—C12	119.01 (15)
C1—N1—Cu	123.47 (11)	N1—C12—C4	122.22 (16)
C12—N1—Cu	116.80 (11)	N1—C12—C11	118.15 (14)
C10—N2—C11	118.80 (15)	C4—C12—C11	119.63 (16)
C10—N2—Cu	132.20 (12)	C1—C13—H13A	109.5
C11—N2—Cu	108.95 (11)	C1—C13—H13B	109.5
N1—C1—C2	120.71 (17)	H13A—C13—H13B	109.5
N1—C1—C13	118.29 (15)	C1—C13—H13C	109.5
C2—C1—C13	121.00 (17)	H13A—C13—H13C	109.5
C3—C2—C1	120.38 (18)	H13B—C13—H13C	109.5
C3—C2—H2A	119.8	C10—C14—H14A	109.5
C1—C2—H2A	119.8	C10—C14—H14B	109.5
C2—C3—C4	119.86 (17)	H14A—C14—H14B	109.5
C2—C3—H3A	120.1	C10—C14—H14C	109.5
C4—C3—H3A	120.1	H14A—C14—H14C	109.5
C12—C4—C3	117.10 (18)	H14B—C14—H14C	109.5
C12—C4—C5	119.28 (18)	C15—O1—Cu	119.93 (13)
C3—C4—C5	123.61 (18)	O2—C15—O1	128.13 (18)
C6—C5—C4	121.48 (18)	O2—C15—H15	118.8
C6—C5—H5A	119.3	O1—C15—H15	112.9
C4—C5—H5A	119.3	C16—O3—Cu	126.20 (12)
C5—C6—C7	120.61 (18)	O4—C16—O3	125.51 (17)
C5—C6—H6A	119.7	O4—C16—H16	118.8
C7—C6—H6A	119.7	O3—C16—H16	114.6
C8—C7—C11	116.55 (17)	Cu—O5—H5B	117.0
C8—C7—C6	123.44 (18)	Cu—O5—H5C	121.6
C11—C7—C6	119.99 (19)	H5B—O5—H5C	107.7
C9—C8—C7	120.23 (18)		

### Hydrogen-bond geometry (Å, °)

**Table:**

*D*—H···*A*	*D*—H	H···*A*	*D*···*A*	*D*—H···*A*
O5—H5C···O2^i^	0.88	1.86	2.714 (2)	166
O5—H5B···O4^ii^	0.89	1.72	2.605 (2)	175

Symmetry codes: (i) −*x*, *y*−1/2, −*z*+1/2; (ii) *x*, *y*−1, *z*.
